# *Escherichia coli* type III secretion system 2: a new kind of T3SS?

**DOI:** 10.1186/1297-9716-45-32

**Published:** 2014-03-19

**Authors:** Mingxu Zhou, Zhiyan Guo, Qiangde Duan, Philip R Hardwidge, Guoqiang Zhu

**Affiliations:** 1 College of Veterinary Medicine, Yangzhou University, Yangzhou 225009, China; 2 Jiangsu Co-Innovation Center for Important Animal Infectious Diseases and Zoonoses, Yangzhou 225009, China; 3 Agriculture College, Weinan Vocational and Technical College, Weinan 714000, China; 4 College of Veterinary Medicine, Kansas State University, 66506 Manhattan, KS, USA

## Abstract

Type III secretion systems (T3SSs) are employed by Gram-negative bacteria to deliver effector proteins into the cytoplasm of infected host cells. Enteropathogenic *Escherichia coli* use a T3SS to deliver effector proteins that result in the creation of the attaching and effacing lesions. The genome sequence of the *Escherichia coli* pathotype O157:H7 revealed the existence of a gene cluster encoding components of a second type III secretion system, the *E. coli* type III secretion system 2 (ETT2). Researchers have revealed that, although ETT2 may not be a functional secretion system in most (or all) strains, it still plays an important role in bacterial virulence. This article summarizes current knowledge regarding the *E. coli* ETT2, including its genetic characteristics, prevalence, function, association with virulence, and prospects for future work.

## Table of contents

1. Introduction

2. Genetic characteristics and differentiation of ETT2

3. Prevalence and distribution of ETT2

4. Putative functions of the ETT2

4.1 ETT2 encodes a functional secretion system?

4.2 ETT2 regulates bacterial virulence

4.3 Association of ETT2 with other virulence factors

5. Conclusions

6. Competing interests

7. Authors’ contributions

8. Acknowledgements

9. References

## 1. Introduction

Many pathogenic Gram-negative bacteria utilize type III secretion systems (T3SSs) to subvert eukaryotic signaling pathways by injecting virulence proteins into the host cell cytoplasm [1-5]. Intracellular pathogens, such as *Salmonella*, *Shigella*, and *Chlamydia*, use T3SS for attachment to and/or invasion of host cells [6-8]. *Yersinia enterocolitica* and *Yersinia pseudotuberculosis* induce macrophage apoptosis and subvert host innate immunity by injecting effectors through the T3SS [9-11]. Enteropathogenic *E. coli* (EPEC) and enterohemorrhagic *E. coli* (EHEC) use the T3SS to deliver effector proteins that result in the creation of the attaching and effacing (A/E) lesions [12,13]. *Salmonella* utilize multiple type III secretion systems, with the first, *Salmonella* pathogenicity islands 1 (SPI-1) T3SS, playing a role in invasion into host cells, while the second, SPI-2 T3SS, is important for intracellular survival [14]. The complete genome sequencing of two EHEC O157:H7 strains, EDL933 and Sakai, revealed the presence of a gene cluster predicted to encode an additional T3SS. This T3SS was designated as the *E. coli* type III secretion system 2 (ETT2), to distinguish it from the locus of enterocyte effacement (LEE) - encoded T3SS, which is now called ETT1 [15,16].

EHEC and other Shiga-like toxin-producing *E. coli* (STEC), including the O157:H7 serotype, are responsible for diseases in humans and animals whose clinical spectrum includes diarrhea, hemorrhagic colitis, and hemolytic uremic syndrome (HUS) [17-20]. There are no proven effective treatments, and administering antibiotics is often contraindicated because they may enhance the progression of enteritis to HUS [21]. While the function of the EHEC ETT1 has been well characterized [22-25], the function of ETT2 and its role in virulence is less clear.

## 2. Genetic characteristics and differentiation of ETT2

**Figure 1:**
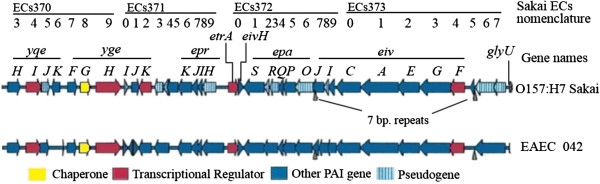
**Structure of ETT2 gene cluster in strain EHEC Sakai and EAEC 042.** The ETT2 gene cluster includes 35 ORFs (ECs3703 – Ecs3737) and is adjacent to the tRNA locus *glyU* on the bacterial chromosome. Frame in yellow indicates gene encoding a chaperone, red indicates gene coding for a transcriptional regulator, blue indicates gene encoding other pathogenicity island (PAI) gene, stripe indicated gene is a pseudogene.

The genes required for ETT1 function, including the T3SS apparatus and the secreted proteins, are encoded on the LEE in EPEC and EHEC. This virulence locus is about 35.4-kb in size and contains 41 open reading frames (ORFs) [24-26]. Up to 32 other non-LEE-encoded effector proteins can also be delivered into host cells by the ETT1 to subvert eukaryotic cell biology [27]. The ETT2 is approximately 29.9-kb in size and is adjacent to the tRNA locus *glyU* on the bacterial chromosome (Figure 1). The ETT2 gene cluster includes at least 35 ORFs, from ECs3703 to ECs3737 in the Sakai strain (GenBank: NC_002695; Table 1). The G + C content of the ETT2 sequence is lower (36.9%) than that of the *E. coli* chromosome (50.8%), suggesting that the ETT2 gene cluster was a pathogenicity island inserted into EHEC O157:H7 strains [28,29].

**Table 1:** Homologs and putative functions of ETT2 ORFs

**Gene in Sakai strain**	**Length (bp)**	Homologs^*^	**Putative function**
ECs3703	693	*yqeH*	
ECs3704	810	*yqeI*	Sensory transducer
ECs3705	480	*yqeJ*	
ECs3706	438	*yqeK*	
ECs3707	492	*ygeF*	
ECs3708	492	*ygeG*,*sicA*	Chaperone
ECs3709	1377	*ygeH*	Transcriptional regulator
ECs3710	219	B2853	
ECs3711	402	B2854, *iagB*	
ECs3712	633	*ygeK*/B2856, *ssrB*	Regulator
ECs3713	432	B2857	
ECs3714	261	B2858, *orgB*	
ECs3715	582	B2859, *orgA*	
ECs3716/*eprK*	735	*prgK*	Lipoprotein precursor
ECs3717/*eprJ*	333	*prgJ*, *mxiI*	
ECs3718/*eprI*	240	*prgI*, *mxiH*	
ECs3719/*eprH*	735	*prgH*, *mxiG*	
ECs3720/*etrA*	501	*ntrC*	Transcriptional regulator
ECs3721/*epaS*	1122	*spaS*	Surface presentation of antigens protein
ECs3722/*epaR2*	237	*spaR*	
ECs3723/*epaR1*	468		
ECs3724/*epaQ*	261	*spaQ*	
ECs3725/*epaP*	666	*spaP*	Surface presentation of antigens protein
ECs3726/*epaO*	987	*spaO*	Surface presentation of antigens protein
ECs3727/*eivJ*	618	*spaN*/*invJ*	
ECs3728	234		
ECs3729/*eivI*	336	*spaM*/*invI*	
ECs3730/*eivC*	1320	*spaI*/*invC*	Invasion protein
ECs3731/*eivA*	2061	*invA*	
ECs3732/*eivE*	1146	*invE*	Invasion protein
ECs3733/*eivG*	1704	*invG*	
ECs3734/*eivF*	750	*invF*	Transcriptional regulator
ECs3735	180	*ydcX*	Inner membrane protein
ECs3736/*pkgA*	1059	B2863	Phosphorylase kinase and glucoamylase
ECs3737	606	B2862	

*yqeHIJK, *ygeFGH*, *ydcX*, B2853, B2354, B2356-B2359, B2362, and B2363 are conserved genes in *E. coli* K-12. *orgA*, *orgB*, *iagB*, *prgHIJK*, *spaOPQRS*, *invA*, *invC*, *invE*, *invF*, *invG*, *invI*, and *invJ* are *Salmonella enterica* SPI-1 genes. *mxiI*, *mxiG* and *mxiH* are *Shigella flexneri* genes. *ntrC* is from *Herbaspirillum seropedicae*.

It has been shown that the ETT2 gene cluster exists in whole or in part in the majority of *E. coli* strains, regardless of their pathogenicity, but with significant variability [29-31]. Sequence alignment analysis revealed that the ETT2 with all 35 ORFs in the Sakai strain also contains some pseudogenes, while EAEC 042 not (Figure 1, [31]). Cheng divided ETT2 variants into 11 isoforms (designated as type A to type K), with type A representing the complete ETT2 island with 35 ORFs [32].

## **3**. Prevalence and distribution of ETT2

The ETT2 gene cluster is primarily associated with STEC strains. Hartleib et al. detected the presence of ETT2 locus in 245 strains of *E. coli*, in 25 strains of *Salmonella enterica*, and in 10 other bacteria, using both PCR and Southern blotting. The ETT2 was distributed ubiquitously among intestinal *E. coli* strains, but not in extra-intestinal (including serotypes O2:H7, O6:H12, O18:H7) or non-pathogenic (including serotypes O5, O74) *E. coli* strains or in other enteric bacteria such as *Salmonella*, *Yersinia*, and *Shigella*[28]. Serotype O55:H7 strains were shown to be the most recent precursors of the virulent O157:H7 EHEC strains [33]. BLAST Genome on NCBI revealed O55:H7 EPEC strains share 49% fragment of the intact ETT2 cluster, losing almost all genes from ECs3711 to ECs3731 (Table 1). Genome analysis also showed that, all whole-genome sequenced EPEC strains (except serotype O127:H6) and STEC (including serotypes both O157 and non-O157) strains carry the ETT2 gene cluster.

ETT2 is prevalent in a majority of characterized human diarrheagenic *E. coli* isolates (54/83; 65%), and all STEC strains tested (except serotype O177) contain the ETT2 gene cluster [34]. However, ETT2 prevalence in uropathogenic *E. coli* (UPEC) strains is low [35].

In animal originated *E. coli* strains, ETT2 is also common. Cheng et al. isolated 92 pathogenic *E. coli* strains from weaned piglets with edema and/or diarrhea and 76 pathogenic *E. coli* strains from dairy cows with clinical or sub-clinical mastitis and detected the ETT2 ORFs by PCR. Most isolates (85.9%) from piglets contained an intact or partial ETT2 gene cluster, significantly higher than those isolated from cows (47.4%) [32]. Prager also identified ETT2 loci in 117 STEC strains originating from piglets suffering from edema disease or colibacillosis [30].

## 4. Putative functions of the ETT2

### 4.1 ETT2 encodes a functional secretion system?

The T3SSs are comprised of a basal body that spans the cell membranes and a needle-like complex that extends from the outer membrane, through which effectors are secreted. The basal body consists of an inner ring, an outer ring, and a neck that spans the periplasm. A functional T3SS requires additional elements, including a cytosolic regulatory complex linked to the basal body, an inner rod that constitutes the base of the needle, and a translocon protein complex that forms a pore in the host membrane [36].

The ETT2 gene cluster was originally regarded as a SPI-1-like island, of which 19 ORFs are highly homologous with the SPI-1 T3SS of *Salmonella enterica* serovar Typhmurium [37,38]. However, some significant differences exist between the *E. coli* ETT2 and SPI-1 T3SS: (i) *spaR* (encoding the T3SS inner ring) is divided into two ORFs, *epaR1* and *epaR2*, in the ETT2 gene cluster, and (ii) *invB* and *invH* are absent from the ETT2 gene cluster. In the *Salmonella* SPI-1 T3SS, InvB is required for secretion and translocation of SopE and SopE2 and for stabilization of SopE2 in the bacterial cytosol [39]. InvH is necessary for localization of InvG to the outer membrane, but InvH is not a component of the type III secretion apparatus [40]. Several important T3SS structural genes, including *prgH* (encoding the inner ring) and *sipABCD* (encoding the needle tip and translocon), are disrupted by frameshifts or are absent [41]. Thus, it appears that this partial ETT2 gene cluster itself does not encode a functional secretion system in EHEC O157 [31].

Nevertheless, even if some necessary genes are missing or nonfunctional in ETT2, it seems possible that genes in ETT1 might complement those gene functions, in order to construct a working apparatus for secretion. Otherwise, *pkgA*, a gene at one end of ETT2 cluster encoding a putative glucoamylase that targets glycogen metabolism within host cell was identified, and speculated to be a ETT2 effector [42]. Another four putative effector genes disrupted by frameshifts in EHEC were found in the EAEC 042 as well, indicating there still has possibility of secretion ability of ETT2 in the future [43].

### 4.2 ETT2 regulates bacterial virulence

However, the ETT2 still plays a crucial role in virulence. Co-regulation between the T3SSs is important to *Salmonella* biology. Normally, effectors encoded in SPI-1 are translocated specifically by the SPI-1 T3SS and effectors encoded in SPI-2 are translocated specifically by the SPI-2 T3SS. Interestingly, sometimes, the *Salmonella* T3SS translocates certain proteins encoded not only in SPI-1, but also outside the locus [44,45]. SPI-2 T3SS could translocate both effectors encoded in SPI-1 and SPI-2. The ETT2 gene cluster can also regulate genes encoded elsewhere on the chromosome.

Zhang et al. targeted the *ygeH* (ECs3709), *etrA* (ECs3720), and *eivF* (ECs3734) genes of the Sakai 813 strain, and demonstrated the negative effects on gene transcription within the LEE by EtrA and EivF: deletion of *etrA* and *eivF* led to increased secretion of proteins encoded by the LEE and to increased adhesion to human intestinal cells [46].

The degenerate ETT2 gene cluster from *E. coli* O157:H7 can not only regulate the expression of virulence-associated genes encoded elsewhere, but also can affect bacterial virulence. Ideses et al. compared the virulence of WT *E. coli* 789 with ETT2 deletion mutants in 1-day-old chickens and found that the ETT2 mutant was substantially attenuated [47]. Yao et al. deleted either the entire ETT2 gene cluster or only *eivA* (ECs3732) gene in the meningitis-causing *E. coli* K1 strain EC10 and observed that the ETT2 deletion mutant exhibited both a 50% reduction in invasion and an 80% decrease in intracellular survival in human brain microvascular endothelial cells (HBMECs) as compared with WT EC10 [48], which indicated that ETT2 is necessary in the pathogenic process of *E. coli* stain K1 interacting with host cells. Sheikh et al. also reported that a *hilA* (encoding a *Salmonella enterica* regulator) homolog, *eilA*, coordinately activated both the EivF, EivA and presumably other effectors encoded elsewhere on the genome (adjacent to tRNA locus *selC*), influencing both bacterial adherence to epithelial cells and biofilm formation, which indirectly suggested the role of ETT2 in bacterial virulence [49,50].

### 4.3 Association of ETT2 with other virulence factors

In the veterinary field, as the ETT2 is primarily associated with EHEC and STEC, it is common to find Stx2e in ETT2 positive strains [32]. In other pathogenic *E. coli* of different O: H serotypes, the ETT2 island is also associated with one or more pathogenicity islands, such as LEE, HPI and LPA [28].

Prager et al. investigated 266 STEC isolates from various human clinical sources and found that different virulence genes were associated with the ETT2. Some genes, including *stx2d* (encoding a Shiga toxin activated by elastase) occur alone or in combinations with ETT2 or LPA, but never with the LEE [51]. It will be important to confirm whether the expression and/or secretion of these virulence factors are affected by the ETT2 island.

## **5**. Conclusions

The ETT2 is prevalent in pathogenic EHEC and STEC strains and is likely important to both human and veterinary medicine. While the exact function of the ETT2 has not been defined clearly, rather than encoding a secretion system, recent work has demonstrated its importance to bacterial adherence and in regulating the expression of other virulence factors, and further study of the ETT2 may help to elucidate details governing bacterial virulence gene regulation. While most serotypes of *E. coli* carry an impaired ETT2 gene cluster, the presence of an intact ETT2 might be used to identify highly-pathogenic EHEC or STEC strains and for molecular fingerprinting of epidemic strains in humans and animals.

## 6. Competing interests

The authors declare that they have no competing interests.

## 7. Authors’ contributions

MZ and ZG collected the data. MZ reviewed the collections and wrote the manuscript. GZ, PRH and QD gave their comments and helped revising this manuscript. All authors read and approved the final manuscript.
